# Improving research and policy interactions requires a better understanding of what works in different contexts

**DOI:** 10.1186/s13584-018-0256-6

**Published:** 2018-10-03

**Authors:** Joshua J. Robinson, Nicholas Mays, Alec Fraser

**Affiliations:** Department of Health Services Research & Policy, London School of Hygiene and Tropical Medicine, 15-17 Tavistock Place, London, WC1H 9SH UK

**Keywords:** Evidence-informed policy, Evidence-based policy, Academic and policy maker collaborations, Research and policy interactions

## Abstract

There is keen interest in many jurisdictions in finding ways to improve the way that research evidence informs policy. One possible mechanism for this is to embed academics within government agencies either as advisers or full staff members. Our commentary argues that, in addition to considering the role of academics in government as proposed by Glied and colleagues, we need to understand better how research and policy interactions function across policy sectors. We believe more comparative research is needed to understand if and why academics from certain disciplines are more likely to be recruited to work in some policy sectors rather than others. We caution against treating government as monolithic by advocating the same model for collaborative interaction between academics and government. Lastly, we contend that contextualized research is needed to illuminate important drivers of research and policy interactions before we can recommend what is likely to be more and less effective in different policy sectors.

## Background

### The ‘evidence revolution’ and the call for more interaction between academics and government policy-makers

The ‘evidence revolution’ to make better use of research in policy has taken root in many countries and policy contexts around the world. In many cases, it has become the publicly expressed expectation, not the exception. For example, in the United Kingdom, this expectation was enshrined nearly two decades ago in government White Papers such as *Modernising Government* and *Professional Policymaking for the Twenty First Century* [1, 2]. Such expectations align with the aspirations of ‘modern’ governments such as transparency, accountability and a less ideologically driven approach to improving public service provision [3–5]. An evidence-based policy (EBP) discourse also serves to legitimize policy-making through, at a minimum, the appearance of objectivity afforded by a government’s commitment to the use of scientific knowledge [5, 6]. Yet, it is well known that direct use of research evidence in policy is difficult to assess, research evidence as a form a knowledge must compete with many other ways of knowing and research evidence is one among many contributors to policy [7]. Recognition that a pure form of EBP is unlikely to be achievable (or even necessarily desirable) has resulted in a shift of position among most advocates of the better use of evidence towards the more modest ambition of ‘evidence-informed’ policy-making (EIP) [8–10].

The relationship between research evidence and policy has been extensively studied in the literature on knowledge mobilization, translation and exchange activities [11–16]. Such work has moved from the more normative to the more empirical to understand how different models of interaction between academics and policymakers aid in improving the role of research for policy, offering more collaborative and interactive solutions such as co-production and knowledge brokering [17]. A common theme across this literature is the idea that improving the relationship between academics and policymakers or practitioners will lead to both production of more relevant research and greater opportunities for research evidence to be used in decision-making. Such collaborative interactions may take place with academics situated outside government, working on government-commissioned research activities, or inside government where academics may hold formal positions as advisors or, for varying periods of time, become civil servants.

Glied, Wittenberg and Israeli offer their perspective on the role of research evidence and academics in government in their article *Research in Government and Academia* [18]. Situating their perspective in the larger shifts of governments’ policy practices, their collective experience from the United States, England and Israel of working in both academia and policy-making provides insight into the role of academics in government as one mechanism to foster more evidence-informed policy. As they traverse the interaction between health policy research and government, Glied and colleagues offer informed perspectives on barriers and drivers for research use in government, how government agendas shape their desires for, and consequently the production of, specific types of research-based knowledge, and mechanisms for considering how to improve the interaction between research and policy by situating academics in formal governmental roles [18].

As they set out how academics in government can serve a multiplicity of different roles, one of the most intriguing and perhaps provocative points is their reflection on the need to reconsider the role of academics in government from carriers or conduits of scientific knowledge and skills to that of active shaper of knowledge and evidence-informed practices for policy. Glied and colleagues contend that academics are able to use their training and expertise within government to bring research evidence to the fore of policy decisions building on their unique understanding of research and, over time, their appreciation of policy-making environments and processes. They raise the question of what the appropriate role of academics in the research and policy relationship is, leaning toward academics in government serving to inform and improve policy-making by facilitating a closer engagement and accessible dissemination of relevant concepts to decision makers and injecting research evidence into more aspects of policy-making. In so doing they raise the question of whether academics should apply their conceptual ‘filters’ and shape research-based knowledge so that it is more usable in situ. Their point is less about changing the role of the academic from dispassionate producer of evidence to positioned advocate and more about appreciating the unique skillsets academics could utilize to assist governments by seeking out, making sense of, and perhaps using research evidence in ways more conducive to policy environments. In so doing, academics in government could, through a variety of activities, bring knowledge from research and related expertise closer to the site of policy decisions so that it may have a greater chance of informing policy [19].

While this way of considering the role of academics in government is useful, Glied and colleagues’ arguments on the interaction between research and policy could be strengthened with further considerations. First, are there characteristics of academics inclined to enter into health policy roles in government that are distinctive compared to other social policy sectors (e.g., nature or type of disciplinary training) and do any differences have an influence on the way in which they contribute to policy-making? Next, much of the work to date on research and policy interactions provides solutions that assume government is monolithic, offering up models of collaboration intended to fit all contexts. Is EIP differentially operationalized across government departments and policy sectors (i.e. to what extent are Glied et al.’s experiences specific to health policy-making)? Finally, in what ways would a contextualized learning of relationships between research evidence generation, research use and its impact on practice inform their analyses?

## Different academic disciplines in government

As evidenced from Glied and colleagues’ experience [18], and those of two of the current authors (Mays and Robinson) who have served in various governmental roles, academics working in health policy in government often, but not always, appear to come from a small range of disciplines (e.g., typically economics and health sciences). In our experience, it is less often the case that academics from the other social sciences such as sociologists, anthropologists, or political scientists, assume government roles as described by Glied and colleagues [18], and even less likely for those from the humanities (e.g. history). While the lack of other social scientists in government is not necessarily problematic, it may speak to a broader level of how certain types of research are more readily accepted within policymaking environments and may be partly a reflection of the nature of work called for to inform health policy issues. It may be representative of the status of certain kinds of particularly quantitative, positivist pursuit of knowledge and assumptions about certain disciplines’ truth claims as qualitative research may, unfairly, be considered less objective that quantitative pursuits [6, 20]. It may also be because academics from certain disciplines find direct counter-parts in the civil service with whom they can work. A large proportion of the analysts within central government in England, for instance, are economists and, accordingly, in our experience, find it relatively easier to work with academic economists than others.

There may be a number of reasons why government would want academics to serve in formal roles, many of which have been previously pointed out by others and echoed by Glied and colleagues [9, 10, 21]. Academics in government may be positioned to deploy their expertise and skills in manners more conducive for research-based knowledge to inform policy processes than if advising from outside of government, but we know little about how academics in government shape policy practices. We know even less about similarities or differences between health policy as compared to, for example, education or environmental policy. A critical questioning of why certain disciplines and forms of knowledge are more readily accepted across areas in government, and reasons for differences, may shed light on these relationships.

## Academic-policy-maker interactions in different policy sectors

While research and policy interactions have been extensively studied in health policy, there is a growing literature on research and policy interactions across policy sectors [9, 17, 22, 23]. Too often in research and policy studies government is treated as monolithic, assuming what happens in one policy sector is broadly applicable to others. Relatively few studies examine, for instance, academic and civil servant collaborations as a unit of analysis to understand how these relationships are enacted and negotiated in practice [14, 19, 24, 25], with a few notable exceptions [26, 27]. We see fewer studies attempting to dissect why similarities and differences in research and policy, and that of academic and civil servant collaborations, exist across policy sectors (e.g., health, education). Empirical work that draws on more diverse theoretical approaches including, but not limited to, the use of policy theory, such as the various strands of institutionalism, and organizational sociology, is needed on the interaction between research and policy to better understand how governments interact with, for example, academics, and what works in context. An excellent example can be seen in Ferlie et al.’s narrative synthesis of the diversity of approaches to knowledge mobilization in the healthcare management literature. The authors highlight an epistemological turn in the evolution in the healthcare knowledge mobilization literature from 2000 signaling a challenge to hierarchical models of evidence based on medical authority in favor of qualitative and narrative forms and a shift from linear conceptualizations of knowledge transfer to relational and organic (trust based) models. A further helpful source – this time based more firmly on the interactions between policy makers and academics is Cairney and Oliver’s work related to the need for better incorporation of policy theories and innovative approaches to both improving and understanding research and policy interactions [18, 19]. These authors highlight the importance of persuasion, emotion, and beliefs as tools for academic researchers to make their work more accessible to policy makers.

## Problematizing the relationship between research evidence generation, use and impact on practice

Frequently, proponents of improving the relationship between research and policy, including many knowledge mobilization, translation and exchange proponents, have approached the interaction from a narrowly conceived ‘use’ perspective [28]. However, viewing the function of research for policy purely through such instrumental terms (i.e., direct use in policy) has begun to give way to a discourse that recognizes that research has other functions such as substantiating policy agendas or shaping policy through the broader ideas derived from research findings [28, 29]. This represents an important shift in conceiving the interaction between research and policy and opens opportunities to investigate this relationship from new perspectives. If research evidence is only one policy puzzle piece, how should that modify our understanding of why government would adopt an EIP discourse and consequentially enact EIP practices such as having academics serve in formal governmental roles?

A deeper understanding of the social function of research and its relationship with policy requires moving beyond the paradigmatic dominance in the knowledge mobilization, translation and exchange literature of an instrumental theory of knowledge use and its concomitant recommendations for collaborative working relationships premised on improving relationships to increase use such as co-production and knowledge brokering. This is not to discredit or minimize the importance of such models for developing closer working relationships between research and policy. Rather, it is to recognize that the pursuit of generalizable models of collaboration may have come at the price of advancing contextualized knowledge of other functions of research in and for policy. Too few studies have attempted to develop alternative frameworks of research and policy interactions that appreciate that research evidence serves multiple functions for policy in addition to direct use of findings in policy [30]. Consequently, we know more about what works in terms of collaborative models to improve the use of research in policy as opposed to the wider drivers of EIP practices – where, when, why and how organizations such as governmental departments adopt, enact and seek to employ EIP practices [19].

## Conclusion

Research and policy interactions are an area deserving of more comparative empirical analysis if we are to ensure research evidence and academics have a seat at the policy-making table. Having academics serve in government is one mechanism for operationalizing evidence-informed policy practices, but we need to know more about where and for what purposes these relationships flourish (or decline). Developing more nuanced appreciations of research and policy interactions involving different disciplines, across different policy sectors is needed before we can suggest which models of interaction might fit different settings best. This also entails understanding what draws academics to work in government, what, if any, characteristics of academics drawn to health policy are unique compared to academics working in other areas of government, and how academics in government influence actual policy practices. To do so, we also need research and policy studies that include understanding the wider reasons why governments pursue EIP.

## Abbreviations

EBP: Evidence-based policy
EIP: Evidence-informed policy
